# Hyperactive mTORC1 disrupts habenula function and light preference in zebrafish model of Tuberous sclerosis complex

**DOI:** 10.1016/j.isci.2024.110149

**Published:** 2024-05-28

**Authors:** Olga Doszyn, Magdalena Kedra, Justyna Zmorzynska

**Affiliations:** 1 Laboratory of Molecular and Cellular Neurobiology, International Institute of Molecular and Cell Biology in Warsaw, 02-109 Warsaw, Poland; 2 Laboratory of Developmental Neurobiology, International Institute of Molecular Mechanisms and Machines, 02-247 Warsaw, Poland

**Keywords:** behavioral neuroscience, Molecular neuroscience, Neurogenetics, neuroscience, sensory neuroscience

## Abstract

Mechanistic target of rapamycin complex 1 (mTORC1) is an integration hub for extracellular and intracellular signals necessary for brain development. Hyperactive mTORC1 is found in autism spectrum disorder (ASD) characterized by atypical reactivity to sensory stimuli, among other symptoms. In Tuberous sclerosis complex (TSC) inactivating mutations in the *TSC1* or *TSC2* genes result in hyperactivation of the mTORC1 pathway and ASD. Here, we show that lack of light preference of the TSC zebrafish model, *tsc2*^*vu242/vu242*^ is caused by aberrant processing of light stimuli in the left dorsal habenula and *tsc2*^*vu242/vu242*^ fish have impaired function of the left dorsal habenula, in which neurons exhibited higher activity and lacked habituation to the light stimuli. These characteristics were rescued by rapamycin. We thus discovered that hyperactive mTorC1 caused aberrant habenula function resulting in lack of light preference. Our results suggest that mTORC1 hyperactivity contributes to atypical reactivity to sensory stimuli in ASD.

## Highlights

• *tsc2* mutants show lack of light preference
• *tsc2* mutants show mTorC1 hyperactivation in the left dorsal habenula
• The left dorsal habenula of *tsc2* mutants exhibit aberrant response to light stimuli
• Rapamycin injections to habenula rescue impaired light-preference

## Graphical abstract

**Figure:**
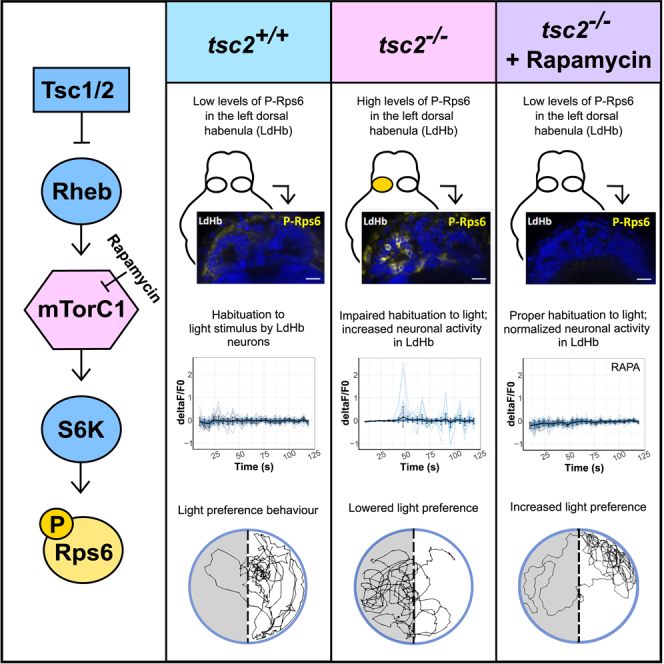

## Introduction

Mechanistic target of rapamycin complex 1 (mTORC1) is an integration hub for extracellular and intracellular signals that controls cell homeostasis by regulating translation, protein degradation, transcription, and cytoskeleton dynamics.^1^ mTORC1 is necessary for proper brain development and coordinates proliferation, migration, differentiation, synaptogenesis, and neuronal activity.^1^ Hyperactivation of mTORC1 is a hallmark of many developmental diseases linked to an increased risk of developing autism spectrum disorder (ASD), including Fragile X syndrome, Angelman syndrome, PTEN-associated ASD or Tuberous sclerosis complex (TSC).^2^^,^^3^^,^^4^^,^^5^ Additionally, dysregulation of mTORC1-dependent signaling has been reported in cases of non-syndromic ASD, both in animal models as well as patients.^6^^,^^7^^,^^8^^,^^9^ ASD has a prevalence of 1% in general population and its symptoms include social deficits, atypical reactivity to sensory stimuli, repetitive behaviors, and speech delay.^10^

TSC is an exemplary genetic disease with mTORC1 hyperactivation. Inactivating mutations in the *TSC1* or *TSC2* genes cause lack of functional TSC1-TSC2 complex and result in hyperactivation of the mTORC1 pathway.^1^ Patients with TSC suffer from epilepsy, benign tumors, and TSC-associated neuropsychiatric disorders (TANDs). TANDs include ASD and do not fully correlate with tumor or seizure burden.^11^ Approximately 40% of the TSC patients develop ASD, which makes TSC the most frequent hereditary cause of ASD.^12^ However, the underlying pathomechanisms of the TSC-associated ASD are still obscure.

Aberrant sensory processing leading to “sensory overload” is a hallmark of ASD,^13^ however, the mechanism underlying this deficit is not fully understood. Individuals with ASD often attempt to avoid visual stimulation^14^ and show high levels of mTORC1 activity.^2^^,^^3^^,^^4^^,^^5^^,^^6^^,^^7^^,^^8^^,^^9^ Here, we investigated light processing in the light preference paradigm in the zebrafish model of TSC^15^ to identify mTorC1 as an underlying cause for aberrant activity of the left dorsal habenula (LdHb) and for atypical response to light in the light-preference test. Our results show that mTORC1 hyperactivity underlies atypical reactivity to light stimuli in TSC and link sensory deficits seen in TSC patients suffering from ASD with hyperactive mTORC1.

## Results

### The *tsc2*^*vu242/vu242*^ mutants lack light preference behavior

**Figure 1:**
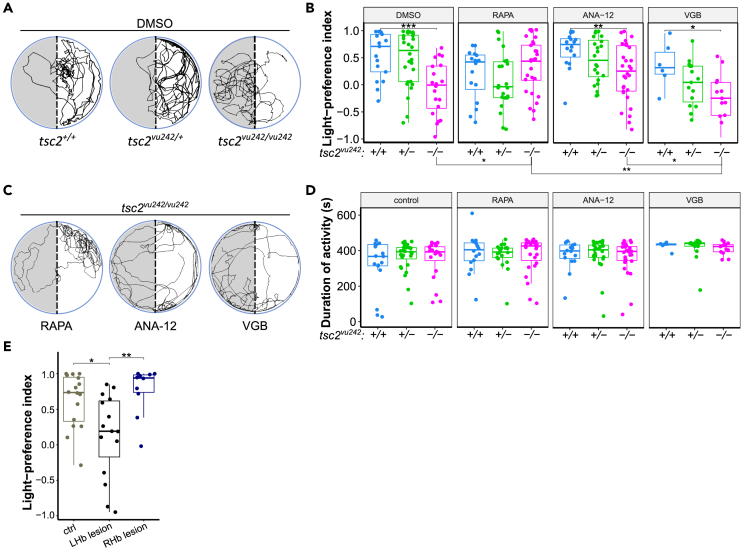
Light-preference test in *tsc2*^*vu242*^ fish (A) Exemplary tracks of *tsc2*^*vu242*^ fish from the light-preference test. (B) Light-preference index of *tsc2*^*vu242*^ fish after various treatments (*tsc2*^*vu242/vu242*^ vs. *tsc2*^*+/+*^: *p* = 0.0009 for DMSO, *p* = 0.588 for rapamycin (RAPA), *p* = 0.005 for ANA-12, *p* = 0.044 for vigabatrin (VGB); for *tsc2*^*vu242/vu242*^ treated with DMSO vs. RAPA *p* = 0.048). The dots on the boxplots represent the number of fish in the experiment (*N* > 10 per experimental group, except VGB *tsc2*^*+/+*^ where *N* = 6). (C) Exemplary tracks of *tsc2*^*vu242/vu242*^ mutant treated with RAPA, ANA-12, or VGB from the light-preference test. (D) Cumulative activity of *tsc2*^*vu242*^ fish during the light-preference test. The dots on the boxplots represent the number of fish in the experiment (*N* > 10 per experimental group, except VGB *tsc2*^*+/+*^ where *N* = 6). (E) Light-preference index of WT fish without lesions and with lesion of left (LHb) or right (Rhb) habenula (unlesioned vs. lesion of the left habenula: *p* = 0.019, lesion of the left habenula vs. lesion of the right habenula: *p* = 0.009). The dots on the boxplots represent the number of fish in the experiment (*N* > 10 per experimental group). See also Figure S1.

It has been long reported that adult zebrafish, presented with a choice between a black and white chamber, exhibit a strong dark preference.^16^ It has also been shown that in a light/gray choice, adult zebrafish prefer the less brightly lit chamber, while in a gray/dark choice, they show no preference, and spend equal amounts of time in both chambers. This avoidance of bright light by the adult zebrafish has been linked to anxiety, as it was attenuated by known anxiolytic compounds. However, in the case of larval zebrafish placed in a light/dark choice, one- and two-week-old fish have shown preference for the light compartment, suggesting that a reversal of the light/dark preference behavior occurs during development. Although the precise mechanism underlying this choice reversal is not known, it has been established that wild-type adult zebrafish show light avoidance, while wild-type larval zebrafish show light preference.^17^ The light-preference assay measures anxiety by comparing times spent in the light and dark compartments. Increased preference for light of zebrafish larvae is indicative of anxiety-like behavior. We have previously shown that the *tsc2*^*vu242/vu242*^ mutant zebrafish exhibit anxiety-like behavior and have elevated cortisol levels.^18^ However, in the light-preference assay, the *tsc2*^*vu242/vu242*^ fish exhibited decreased light preference compared to wild-type (WT) siblings, which strongly preferred the light compartment (Figures 1A and 1B). The total time moving was similar among genotypes (Figure 1D). The lack of light preference of the *tsc2*^*vu242/vu242*^ fish was not prevented by pretreatment with anxiolytic drug ANA-12 (Figures 1B, 1C, and S1A). We have shown before that ANA-12 rescued anxiety-like behaviors of the *tsc2*^*vu242/vu242*^ fish.^18^ Thus, the lack of light preference was not likely to be associated with anxiety. Moreover, the *tsc2*^*vu242/vu242*^ mutants did not have major developmental defects (Figure S1B) or visual problems as they responded to light changes similarly as siblings in the sudden-light-changes assay (Figures S1C‒S1E). The lack of preference for light of the *tsc2*^*vu242/vu242*^ fish was also not changed by treatment with an anti-epileptic drug vigabatrin (VGN) (Figures 1B and 1C). Interestingly, the pretreatment with a direct mTorC1 inhibitor rapamycin reversed the aberrant light response of the *tsc2*^*vu242/vu242*^ mutants in the light-preference test (Figures 1B and 1C). These results led us to the hypothesis that hyperactive mTorC1 underlies the lack of light-preference of the *tsc2*^*vu242/vu242*^ fish.

### Neurons in the left dorsal habenula of *tsc2*^*vu242/vu242*^ mutants show mTorC1 hyperactivation and aberrant response to light stimuli

**Figure 2:**
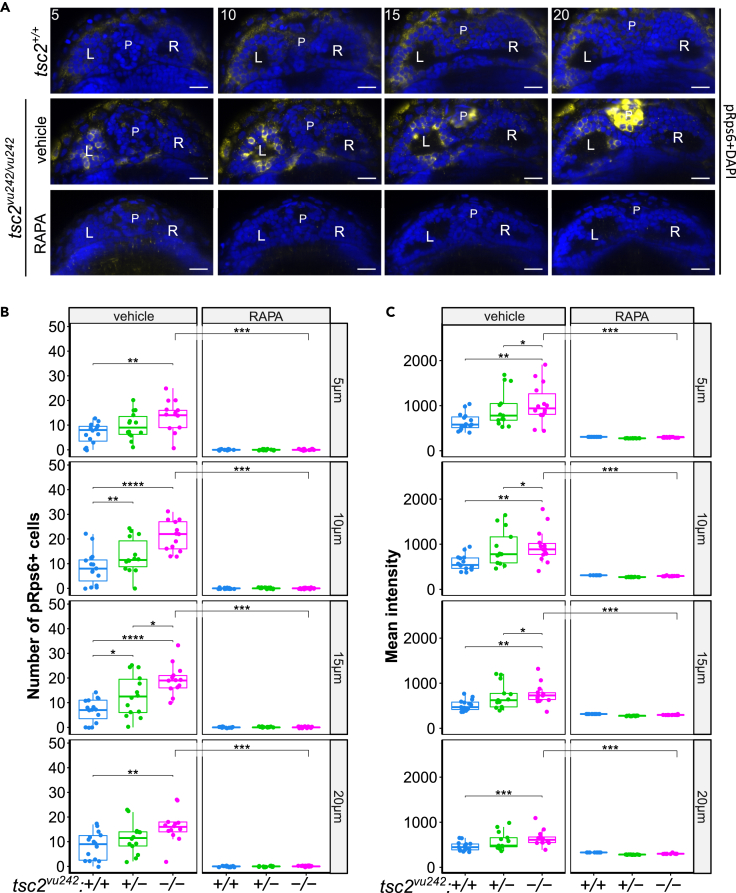
mTorC1 activation in *tsc2*^*vu242*^ fish habenulas (A) Representative optical sections through habenula of *tsc2*^*vu242/vu242*^ fish and their wild-type *tsc2*^*+/+*^ siblings at 5, 10, 15, and 20 μm from the top. pRps6—yellow, nuclei—blue. L—left habenula, R—right habenula, P—pineal complex. Scale bars, 20 μm. (B) Number of pRps6-positive cells in LdHb of *tsc2*^*vu242*^ fish (5 μm: *tsc2*^*vu242/vu242*^ control vs. *tsc2*^*+/+*^ control *p* = 0.004 and vs. *tsc2*^*vu242/vu242*^ treated with RAPA *p* = 1.038 × 10^−5^; 10 μm: *tsc2*^*vu242/vu242*^ control vs. *tsc2*^*+/+*^ control *p* = 4.656 × 10^−5^ and vs. *tsc2*^*vu242/vu242*^ treated with RAPA *p* = 1.038 × 10^−5^; 15 μm: *tsc2*^*vu242/vu242*^ control vs. *tsc2*^*+/+*^ control *p* = 1.584 × 10^−5^ and vs. *tsc2*^*vu242/vu242*^ treated with RAPA *p* = 1.038 × 10^−5^; 20 μm: *tsc2*^*vu242/vu242*^ control vs. *tsc2*^*+/+*^ control *p* = 0.004 and vs. *tsc2*^*vu242/vu242*^ treated with RAPA *p* = 1.038 × 10^−5^). The dots on the boxplots represent the number of fish in the experiment (*N* > 10 per experimental group). (C) Quantification of mean intensity of pRps6 fluorescence from LdHb of *tsc2*^*vu242*^ fish (5 μm: *tsc2*^*vu242/vu242*^ control vs. *tsc2*^*+/+*^ control *p* = 0.0051 and vs. *tsc2*^*vu242/vu242*^ treated with RAPA *p* = 1.0912 × 10^−5^; 10 μm: *tsc2*^*vu242/vu242*^ control vs. *tsc2*^*+/+*^ control *p* = 0.0017 and vs. *tsc2*^*vu242/vu242*^ treated with RAPA *p* = 1.0912 × 10^−5^; 15 μm: *tsc2*^*vu242/vu242*^ control vs. *tsc2*^*+/+*^ control *p* = 0.001 and vs. *tsc2*^*vu242/vu242*^ treated with RAPA *p* = 1.0912 × 10^−5^; 20 μm: *tsc2*^*vu242/vu242*^ control vs. *tsc2*^*+/+*^ control *p* = 0.0007 and vs. *tsc2*^*vu242/vu242*^ treated with RAPA *p* = 1.0912 × 10^−5^). The dots on the boxplots represent the number of fish in the experiment (*N* > 10 per experimental group).

The lack of light preference of the *tsc2*^*vu242/vu242*^ fish, otherwise exhibiting increased anxiety-like behaviors, can be indicative of impaired sensory processing of the light stimulus. Habenula integrates various stimuli and regulates light-preference behavior.^19^^,^^20^^,^^21^ We also confirmed that impairment of LdHb results in a lack of light preference in WT zebrafish (Figures 1E and S1F). To test for mTorC1 hyperactivation in habenulae of the *tsc2*^*vu242/vu242*^ mutants, we checked phosphorylation levels of mTorC1 downstream target: ribosomal protein s6 (Rps6). We found that the number of cells positive for phosphorylated Rps6 (pRps6) in the *tsc2*^*vu242/vu242*^ mutants was increased specifically in the left dorsal habenula (LdHb) compared to WT siblings (Figures 2A and 2B). Also, the pRps6 intensity levels per cell were higher in the *tsc2*^*vu242/vu242*^ LdHb neurons than in the WT fish (Figure 2C). The pRps6 levels were decreased after rapamycin pretreatment in both *tsc2*^*vu242/vu242*^ and WT siblings (Figures 2A–2C).

**Figure 3:**
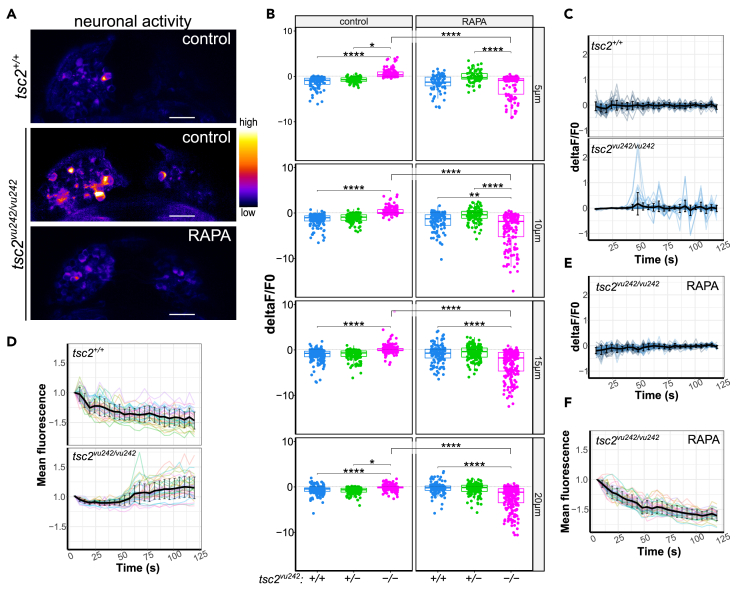
Neuronal activity in LdHb of *tsc2*^*vu242*^ fish (A) Representative images of neuronal activity in the habenulae of *tsc2*^*vu242*^ fish at 10 μm from the top. Scale bars, 20 μm. (B) Cumulative activity of the *tsc2*^*vu242*^ LdHb for all the layers of LdHb (5 μm: *tsc2*^*vu242/vu242*^ control vs. *tsc2*^*+/+*^ control *p* = 6.528 × 10^−33^ and vs. *tsc2*^*vu242/vu242*^ treated with RAPA *p* = 5.958 × 10^−34^; 10 μm: *tsc2*^*vu242/vu242*^ control vs. *tsc2*^*+/+*^ control *p* = 1.1232 × 10^−44^ and vs. *tsc2*^*vu242/vu242*^ treated with RAPA *p* = 1.625 × 10^−47^; 15 μm: *tsc2*^*vu242/vu242*^ control vs. *tsc2*^*+/+*^ control *p* = 5.928 × 10^−31^ and vs. *tsc2*^*vu242/vu242*^ treated with RAPA *p* = 4.68 × 10^−52^; 20 μm: *tsc2*^*vu242/vu242*^ control vs. *tsc2*^*+/+*^ control *p* = 1.104 × 10^−14^ and vs. *tsc2*^*vu242/vu242*^ treated with RAPA *p* = 5.958 × 10^−34^). Dots on boxplots represent single cells. (C) Neuronal activity change over time in the *tsc2*^*vu242*^ LdHb at 10 μm from the top. (D) Normalized mean GCaMP5F fluorescence over time in the *tsc2*^*vu242*^ LdHb at 10 μm from the top. (E) Neuronal activity change over time in the *tsc2*^*vu242/vu242*^ mutant’s LdHb at 10 μm from the top after RAPA pretreatment. (F) Normalized mean GCaMP5F fluorescence over time in the *tsc2*^*vu242/vu242*^ mutant’s LdHb at 10 μm from the top after RAPA. (C-F) The mean with SD is shown in black. 7 fish per genotype per treatment were analyzed (*N* = 7 per experimental group). See also Figure S2.

LdHb contains light-responsive neurons and is responsible for mediating light-preference behavior in zebrafish larvae.^19^^,^^21^ We performed 3D time-lapse imaging of the activity of LdHb neurons in *Tg*(*HuC:GCaMP5G*);*tsc2*^*vu242*^ expressing GCaMP5G calcium indicator under neuron-specific promoter. The single-cell analysis revealed that neuronal activity in LdHb was increased in *tsc2*^*vu242/vu242*^ compared to WT siblings (Figures 3A–3D). With analysis of neuronal activity dynamics across time, we discovered that the activity of light-responsive neurons in the most dorsal layers increased after light stimulation in the WT LdHb but decreased over time, indicative of habituation to constant light stimulus. In contrast, the activity of neurons in the *tsc2*^*vu242/vu242*^ LdHb was lower at the initial stimulation but increased over time (Figures 3C, 3D, and S2). Rapamycin pretreatment normalized neuronal activity of the *tsc2*^*vu242/vu242*^ LdHb (Figures 3E, 3F, and S2) implicating mTorC1 hyperactivation in the aberrant LdHb activity.

### mTorC1 hyperactivation in the left dorsal habenula causes lack of light-preference behavior of the *tsc2*^*vu242/vu242*^ fish

**Figure 4:**
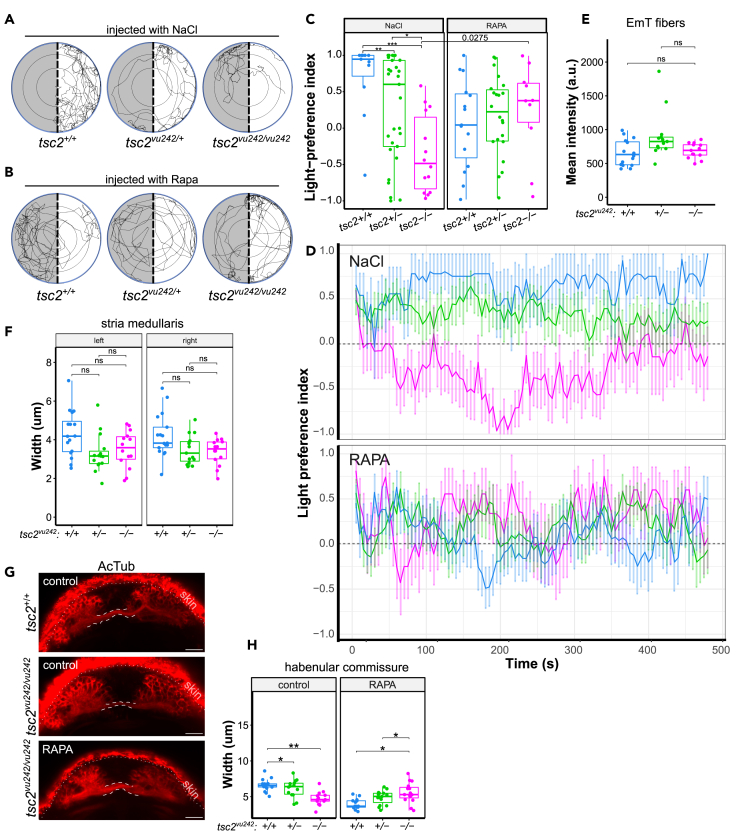
Rapamacin injections to the left habenula and afferent connectivity of LdHb in *tsc2*^*vu242*^ fish (A) Exemplary tracks of the *tsc2*^*vu242*^ fish injected to the left habenula with NaCl from the light-preference test. (B) Exemplary tracks of the *tsc2*^*vu242*^ fish injected to the left habenula with 1 μM rapamycin (Rapa) from the light-preference test. (C) Light-preference index of the *tsc2*^*vu242*^ fish after injections to the left habenula (*tsc2*^*vu242/vu242*^ vs. *tsc2*^*+/+*^: *p* = 0.00015 and vs. *tsc2*^*+/−*^: *p* = 0.006 for NaCl, *p* = *ns* for rapamycin (RAPA); *tsc2*^*vu242/vu242*^ fish injected with RAPA vs. NaCl: *p* = 0.0275). The dots on the boxplots represent the number of fish in the experiment (*N* > 10 per experimental group). (D) Analysis of light-preference index over time of the *tsc2*^*vu242*^ fish after injections to the left habenula (data presented as mean ± SD). (E) Mean intensity of calretinin fluorescence in the left habenulae of the *tsc2*^*vu242*^ fish. The dots on the boxplots represent the number of fish in the experiment (*N* > 10 per experimental group). (F) Width of SM in the *tsc2*^*vu242*^ fish brains. The dots on the boxplots represent the number of fish in the experiment (*N* > 10 per experimental group). (G) Representative horizontal optical sections through HC (outlined) of the *tsc2*^*vu242*^ fish (projection of 8 z-stacks). Scale bars, 20 μm. (H) HC width of the *tsc2*^*vu242*^ fish (*tsc2*^*vu242/vu242*^ vs. *tsc2*^*+/+*^: *p* = 0.002 for DMSO, *p* = 0.011 for RAPA). The dots on the boxplots represent the number of fish in the experiment (*N* > 10 per experimental group). See also Figure S3.

To address further the involvement of hyperactive mTorC1 in LdHb in controlling light preference behavior of the *tsc2*^*vu242/vu242*^ mutants, we microinjected rapamycin directly into the left habenula to locally inhibit mTorC1. The singular injection at 4 dpf did not change mTorC1 activation levels at 5 dpf (Figure S3A), thus we injected rapamycin on two consecutive days, at 3 dpf and 4 dpf (Figures S3B and S3C) and then we tested the injected fish for light preference at 5 dpf. The vehicle-injected *tsc2*^*vu242/vu242*^ fish showed lack of light preference behavior similarly to uninjected mutants and rapamycin injection rescued this impairment (Figures 4A–4D and S3). The *tsc2*^*vu242*^ fish showed normal habenula morphology after injections and similar duration of activity during the light-preference test (Figures S3C and S3D). Analysis of fish activity over time showed that the light-preference index of the *tsc2*^*vu242/vu242*^ mutants decreases over time during first 2 min of analysis (Figure 4D) indicating that they have high preference for dark compartment or aversion to light. After 200 s, the light-preference index of the *tsc2*^*vu242/vu242*^ fish increased slightly but still stayed at the negative level (Figure 4D). This impairment was rescued by rapamycin injection into the LdHb.

The left habenula receives afferent inputs from the eminentia thalami (EmT), the pallium through stria medullaris (SM), and from the right habenula through the habenula commissure (HC).^19^ Therefore, we investigated the development of these afferents in the *tsc2*^*vu242/vu242*^ mutants as their alterations could facilitate impaired LdHb function. The EmT fibers that innervate LdHb are calretinin-positive, thus, we checked anti-calretinin immunofluorescence in the LdHb in whole-mount brain preparations. We determined that the mean intensity of the anti-calretinin signal was similar across genotypes (Figure 4E), suggesting proper innervation of LdHb by EmT in the *tsc2*^*vu242/vu242*^ fish. The immunofluorescence staining against acetylated tubulin (AcTub) revealed that the lateral input to habenulae through SM was not significantly changed in *tsc2*^*vu242/vu242*^ compared to WT (Figure 4F). However, the HC was thinner in the *tsc2*^*vu242/vu242*^ fish compared to the WT siblings, and pretreatment with rapamycin reversed this to its proper width (Figures 4G and 4H) indicating that the right habenula input to LdHb may be involved in the LdHb aberrant function.

## Discussion

We have shown that aberrant activity of the LdHb neurons was associated with hyperactivation of the mTorC1 pathway and decreased light preference in the *tsc2*^*vu242/vu242*^ mutants. The involvement of mTORC1 in neuronal activity is well documented and hyperactive mTORC1 consistently produces neuronal hyperexcitability and seizures.^22^ The increased neuronal activity of LdHb neurons in *tsc2*^*vu242/vu242*^ can be indicative of decreased activation threshold which is seen in the pallium of *tsc2*^*vu242/vu242*^ and is responsible for seizures.^18^ However, anti-epileptic VGB did not rescue light-preference behavior and rapamycin did reverse both, increased neuronal activity of LdHb neurons and light-preference behavior in the *tsc2*^*vu242/vu242*^ fish, suggesting that LdHb activity is not induced by seizures. Instead, hyperactive mTorC1 causes aberrant LdHb function and impairs sensory integration resulting in the lack of light preference of the *tsc2*^*vu242/vu242*^ fish. LdHb integrates light stimuli from EmT and the right habenula with other inputs to produce light-preference behavior. In older zebrafish larvae, deactivation of LdHb by botulinum toxin decreased light preference, and activation of LdHb by optogenetic approach resulted in a preference for light in the WT zebrafish.^21^ We also confirmed that deactivation of the left habenula decreases preference for light in the WT fish. In the *tsc2*^*vu242/vu242*^ fish, however, the LdHb activity is impaired—low at initial stimulation, but increasing in time. It suggests that the threshold for activation may be higher but results in higher neuronal activity when crossed or that the habituation to the light stimulus is impaired. This in turn may cause aversion to the light stimulus and result in lack of light-preference behavior. It is possible that intracellular signaling pathways are abnormally functioning due to hyperactive mTorC1 and therefore the synaptic inputs to LdHb in the *tsc2*^*vu242/vu242*^ fish are not integrated properly or timely.

However, caution should be taken when administering rapamycin or rapamycin derivatives (sirolimus and everolimus) to ASD patients without diagnosed genetic mutation linked to mTORC1 hyperactivity and we suggest that the levels of mTORC1 activation should be checked beforehand as rapamycin may exert some toxic effects. We have observed that after rapamycin treatment, WT fish loose the light preference while the *tsc2*^*vu242/vu242*^ mutant fish show restored light-preference behavior. Rapamycin was previously reported to have deleterious effects on WT animals.^23^ Rapamycin treatment resulted in highly exaggerated neuronal activity induced by kainic acid and induced brain morphology changes in WT rats.^24^ Also in patients, rapamycin derivatives (sirolimus and everolimus), which are often used in cancer or after transplantation, exerted adverse effects when administered systemically.^25^^,^^26^^,^^27^ Consistently with these data, our results also show smaller habenulas after treatment with rapamycin. However, the loss of light preference of WT fish after rapamycin pretreatment may not have been associated with habenula function as the neuronal activity of habenular neurons was not distinguishable from WT fish treated with vehicle. Our results strongly link atypical reactivity to light with hyperactive mTorC1 and suggest that rapamycin derivatives can be used to prevent atypical reactivity to sensory stimuli in ASD with hyperactivated mTorC1.

### Limitations of the study

Modeling human diseases in zebrafish offers numerous advantages due to their genetic similarity and ease of manipulation, however, there are also limitations to consider. Zebrafish have a simpler nervous system and behavioral repertoire compared to humans. While they can display basic behaviors like response to light or movement, studying complex cognitive or emotional behaviors that are relevant to human psychiatric disorders such as ASD is challenging. Zebrafish may also metabolize drugs differently than humans, which can affect the efficacy and toxicity of potential treatments studied in zebrafish models. This can complicate translation of findings to human clinical trials. Zebrafish embryos develop externally, which allows for easier observation of development but the placental environment may serve a very important role in fetal brain development concerning ASD and TSC. Many human diseases are multifaceted and involve interactions between genetic, environmental, and lifestyle factors. Zebrafish models may oversimplify these interactions due to their controlled laboratory environment. Despite these limitations, zebrafish remain a valuable model organism for studying certain aspects of human disorders, especially in areas like developmental biology, genetics, and basic biological processes. Complementary studies using other model organisms and human cell-based assays are needed to validate findings and bridge the gap between zebrafish research and clinical applications.

## STAR★Methods

### Key resources table

**Table:** 

REAGENT or RESOURCE	SOURCE	IDENTIFIER
**Antibodies**		

Mouse monoclonal anti-acetyl-Lys40-Tubulin	GeneTex	Cat# GTX16292; RRID:AB_2887530
Rabbit polyclonal anti-Calretinin	SWant	Cat# 7697; RRID:AB_2721226
Rabbit polyclonal anti-pRps6 (Ser235/236)	Cell Signaling Technology	Cat# CS4858; RRID:AB_916156
Goat anti-Mouse IgG (H+L) Cross-Adsorbed Secondary Antibody, Alexa Fluor™ 568	Thermo Fisher Scientific	Cat# A-11004; RRID:AB_2534072
Goat anti-Rabbit IgG (H+L) Cross-Adsorbed Secondary Antibody, Alexa Fluor™ 488	Thermo Fisher Scientific	Cat# A-11008; RRID:AB_143165

**Chemicals, peptides, and recombinant proteins**		

ANA-12	Sigma-Aldrich/Merck	Cat# SML0209
Dimethyl sulfoxide	Sigma-Aldrich/Merck	Cat# D8418
E3 medium (for neuronal *in vivo* models): NaCl (sodium chloride) KCl (potassium chloride) CaCl2 (calcium chloride) MgCl2 (magnesium chloride)	Chempur	Cat# 117941206 Cat# 117397402 Cat# 118748703 Cat# 116120500
Formaldehyde	Sigma-Aldrich/Merck	Cat# 47608
Glycerol	Sigma-Aldrich/Merck	Cat# G5516
Goat serum	Sigma-Aldrich/Merck	Cat# G9023
Heparin sodium salt from porcine intestinal mucosa	Sigma-Aldrich/Merck	Cat# H3149
Phenol Red	Sigma-Aldrich/Merck	Cat# P0290
Rapamycin	Sigma-Aldrich/Merck	Cat# 553210
RT PCR Mix EvaGreen®	A&A Biotechnology	Cat# 2008
TopVision low melting point agarose	Thermo Fisher Scientific	Cat# R0801
Tricaine	Sigma-Aldrich/Merck	Cat# E10521
Triton™ X-100	Sigma-Aldrich/Merck	Cat# T8787
Tween®20	Sigma-Aldrich/Merck	Cat# P1379
Vigabatrin	Sigma-Aldrich/Merck	Cat# V8261

**Deposited data**		

Raw and analyzed data	This paper	N/A

**Experimental models: Organisms/strains**		

Zebrafish: mixed wild-type strain AB/ TL	Zebrafish Core Facility, International Institute of Molecular and Cell Biology in Warsaw	ZFIN:ZDB-GENO-031202-1
Zebrafish: Tg(HuC:GCaMP5G);tsc2^vu242/+^	Kedra et al.	N/A
Zebrafish: tsc2^vu242/+^	Kim et al.	RRID:ZFIN_ZDB-GENO-180906-3
Zebrafish: tsc2^vu242/ vu242^	Kim et al.	RRID:ZFIN_ZDB-GENO-190115-9

**Oligonucleotides**		

Primer *tsc2* for HRM genotyping, Forward: GAGACCTGCCTGGACATGAT	Doszyn et al.^28^	N/A
Primer *tsc2* for HRM genotyping, Reverse: CTTGGGCAGAGCAGAGAAGT	Doszyn et al.^28^	N/A

**Software and algorithms**		

Fiji	Schindelin et al.^29^	https://fiji.sc/
R 4.3.2	R Foundation for Statistical Computing	https://www.R-project.org
RStudio	Posit Software, PBC	https://posit.co/download/rstudio-desktop/
ZebraLab version 3.22	ViewPoint Behavior Technology	https://www.viewpoint.fr/product/zebrafish/fish-behavior-monitoring/zebralab
ZEN2014 SP1 (black edition)	Zeiss	https://www.micro-shop.zeiss.com/en/us/softwarefinder/software-categories/zen-black

**Other**		

Cokin CREATIVE light-absorbing photographic filters: Neutral grey ND4 – 0.6, Medium size Neutral grey ND8 – 0.9, Medium size	Cokin	Cat# P153 Cat# P154
Borosilicate glass capillaries 0.75 mm inner diameter	Sutter Instruments	Cat# BF150-75-10
Lightsheet Z.1 microscope	Zeiss	https://www.zeiss.com/microscopy/en/products/light-microscopes/light-sheet-microscopes.html
ZebraBox	ViewPoint Behavior Technology	https://www.viewpoint.fr/product/zebrafish/fish-behavior-monitoring/zebrabox

### Resources availability

#### Lead contact

Further information and requests for resources and reagents should be directed to and will be fulfilled by the Lead Contact, Justyna Zmorzynska (j.zmorzynska@imol.institute).

#### Materials availability

This study did not generate new unique reagents. The fish mutant and transgenic lines are protected under material transfer agreement with the institutions that generated the lines. Upon appropriate agreement with these institutions, they can be requested from the lead contact.

#### Data and code availability

• Data: All data is included in the manuscript. Microscopy data reported in this paper will be shared by the lead contact upon request.
• Code: This paper does not report original code.
• Additional information: Any additional information required to reanalyze the data reported in this paper is available from the lead contact upon request.

### Experimental model and study participant details

#### Zebrafish breeding and genotyping

The following lines were used: *tsc2*^*vu242/+*^,^15^ wild-type (mixed strain AB x TL), and *Tg(HuC:GCaMP5G);tsc2*^*vu242/+*^.^18^^,^^30^ Adult and larval zebrafish were bred according to international standards. Zebrafish larvae used in this study were up to 5 dpf and had no specified sex yet. All experiments performed were conducted in accordance with the Act of 15 January 2015 on the protection of animals used for scientific and educational purposes, Directive 2010/63/EU of the European Parliament and of the Council of 22 September 2010 on the protection of animals used for scientific purposes and were approved by the Animal Welfare Commission of the IIMCB. The larvae were genotyped by PCR and high resolution melting technique using primers listed in the key resources table.^18^^,^^28^ Genotyping was performed after collection of behavioral or imaging data in live experiments or after collection of zebrafish heads for immunofluorescence (the tails were used for genotyping). Offspring of at least two parental pairs were used in each experiment.

#### Drug treatments

To prepare stock solutions, drugs were dissolved in E3 (5 mM NaCl, 0.17 mM KCl, 0.33 mM CaCl_2_, 0.33 mM MgSO_4_) or dimethylsulfoxide (DMSO) and were further diluted in E3 or in NaCl (for injections into the habenula). Drugs were administered directly into E3 with the same number of dechorionated fish. Treatments included: 200 nM rapamycin (Sigma-Aldrich/Merck, #553210) from 2 days post-fertilization (dpf), 50 nM ANA-12 or 60 μM VGN (both from Sigma-Aldrich/Merck, Darmstadt, Germany) 24 hours before the behavioral test. For injections into the habenula, 1 μM rapamycin was used.

### Method details

#### Light-preference assay

Light-preference test was performed using live monitoring system Zebrabox (Viewpoint). One fish at a time was tracked in a Petri dish with half of a dish covered with light-absorbing photographic filters (ND4+ND8, Cokin.com) to produce darkness. Tracking was done for 8 min.^18^ Lack of movement of the *tsc2*^*vu242/vu242*^ mutants was mapped to non-motor seizures before,^18^ thus, not moving fish were excluded from the analysis. The light preference index was calculated as cumulative time spent in the dark compartment subtracted from cumulative time spent in the light compartment and divided by the total time of movement ((L-D)/(L+D)) as previously.^21^

#### Habenula lesion

A borosillicate glass capillary (0.75 mm inner diameter, Sutter Instruments) was heat-pulled to obtain a micropipette with an open tip of ∼15 μm. At 4 dpf, fish were sedated with 0.01% Tricaine (E10521, Sigma-Aldrich) and immobilized in 1% low-melting point agarose (Thermo Fisher Scientific) with head facing up. The habenulae were lesioned with the micropipette under a microscope to visualize the region with high accuracy. After 3-4 hours, the fish were freed from agarose and moved to E3 medium without Tricaine. At 5 dpf, the fish were tested for light preference and then fixed in 4% formaldehyde for habenula morphology analysis.

#### Whole-mount immunofluorescence

Heads of zebrafish larvae were fixed in 4% formaldehyde at 5 dpf (overnight at 4°C). Samples were bleached in freshly prepared 3% KOH, 1% H_2_O_2_ solution, permeabilized with PBS containing 0.2% Triton X-100, 20% DMSO, and 0.1% Tween-20, blocked with 3% bovine serum albumin and 10% goat serum, incubated with primary antibodies (anti-pRps6 antibody (Ser235/236), 1:200, CS4858, Cell Signaling Technology, RRID:AB_916156; anti-calretinin, 1:400, CR7697, Swant, RRID:AB_2721226; anti-acetyl-Lys40-tubulin, 1:100, GTX16292, Genetex, RRID:AB_2887530) in PBS-Hep solution (PBS suplemented with 0.2% Tween-20 and 10 μg/ml heparin) at room temperature for 72 hours, washed overnight with PBS-Hep, incubated with secondary antibodies (goat anti-mouse Alexa Fluor 568, 1:1000, or goat anti-rabbit, Alexa Fluor 488, 1:1000, both from Thermo Fisher Scientific) in PBS-Hep for 48 hours, washed again, and mounted.

#### Microinjections into the habenula

A borosillicate glass capillary (0.75 mm inner diameter, Sutter Instruments) was heat-pulled to obtain a micropipette with an open tip of ∼3 μm. The drop was set to 0.5 nL. At 3 and 4 dpf, fish were sedated with 0.01% Tricaine (E10521, Sigma Aldrich) and immobilized in 1% low-melting point agarose (Thermofisher Scientific) with head facing up. Then the solutions (0.9% NaCl or 1 μM rapamycin, containing 0.05% Phenol Red (Sigma-Aldrich) for visualization) were microinjected into the left habenula – the micropipette was inserted between the habenula and the blood vessel surrounding it. 4 drops were administered with 20 sec intervals. After 3-4 hours, the fish were freed from agarose and moved to E3 medium without Tricaine. At 5 dpf, the fish were tested for light preference and then fixed in 4% formaldehyde for whole-mount immunofluorescence.

#### Imaging

Images were acquired using a Zeiss Lightsheet Z.1 microscope (40× water immersion objective, NA = 1.3) at 1024 × 1024 pixel-resolution. Z-stacks of the images were taken with an interval of 0.5 μm for fixed samples and 1 μm for live imaging. The 3D time-lapse images were recorded every 5s for 2 min. at 28.5°C at 4 dpf. Time-lapse images included both habenulae and had an approximate range of 100 μm.

### Quantification and statistical analysis

#### Image analyses

Image analyses were performed using Fiji software. For pRps6, AcTub, and calretinin measurements, regions of interest (ROIs) were drawn manually and cell size, fluorescence intensity, or fiber width were measured using the measurement tool. For neuronal activity analysis, drift correction was performed using the Tischer script embedded in the Fiji software, ROIs were applied manually, measured using the measurement tool in Fiji, and then the fluorescence intensity and derivative (deltaF/F0, where F0 is the minimal intensity value of the cell) were calculated with Rstudio (cran.r-project.org; rstudio.com). All LdHb neurons from 7 fish per genotype per treatment were included in the analysis.

#### Statistical analysis

No predetermination of sample sizes was performed because the number of each genotype could not be predicted due to the random distribution and the early lethality of homozygotes (as previously reported^18^). Equality of variance and normality of residuals were assessed using Levene’s test and Shapiro-Wilk test, respectively. Because these assumptions were not met, data were analyzed by Kruskal-Wallis test with *post-hoc* Wilcoxon test to correct for multiple comparisons. The adjusted *p* < 0.05 was considered statistically significant. Data are presented as medians using boxplots with dots representing data points. Data points outside of boxplots and their whiskers are outliers. In majority of analyses, number of dots represent number of animals included (1 dot is 1 fish) – except for single-cell calcium imaging analysis where 7 fish per genotype per treatment were included. Adjusted p-values are reported on figures with ∗ for *p* < 0.05, ∗∗ for *p* < 0.01, ∗∗∗ for *p* < 0.001, and ∗∗∗∗ for *p* < 0.0001. In most cases, randomization and blinding was assured by lack of knowledge about the genotypes while performing the experiments or collecting the fish for immunofluorescence. All data were analyzed with Rstudio.

## Supplemental information

Document S1. Figures S1‒S3
