# Supplementary material for: Hyperactive mTORC1 disrupts habenula function and light preference in zebrafish model of Tuberous sclerosis complex
Source: iScience. 2024 May 28;27(6):110149. doi: 10.1016/j.isci.2024.110149 (PMC11214417; doi:10.1016/j.isci.2024.110149)
Supplement: Document S1. Figures S1‒S3 [file mmc1.pdf]

iScience, Volume *27*

Supplemental information

Hyperactive mTORC1 disrupts habenula function and light preference in zebrafish model of Tuberous sclerosis complex

Olga Doszyn, Magdalena Kedra, and Justyna Zmorzynska

Supplementary Figures 1-4

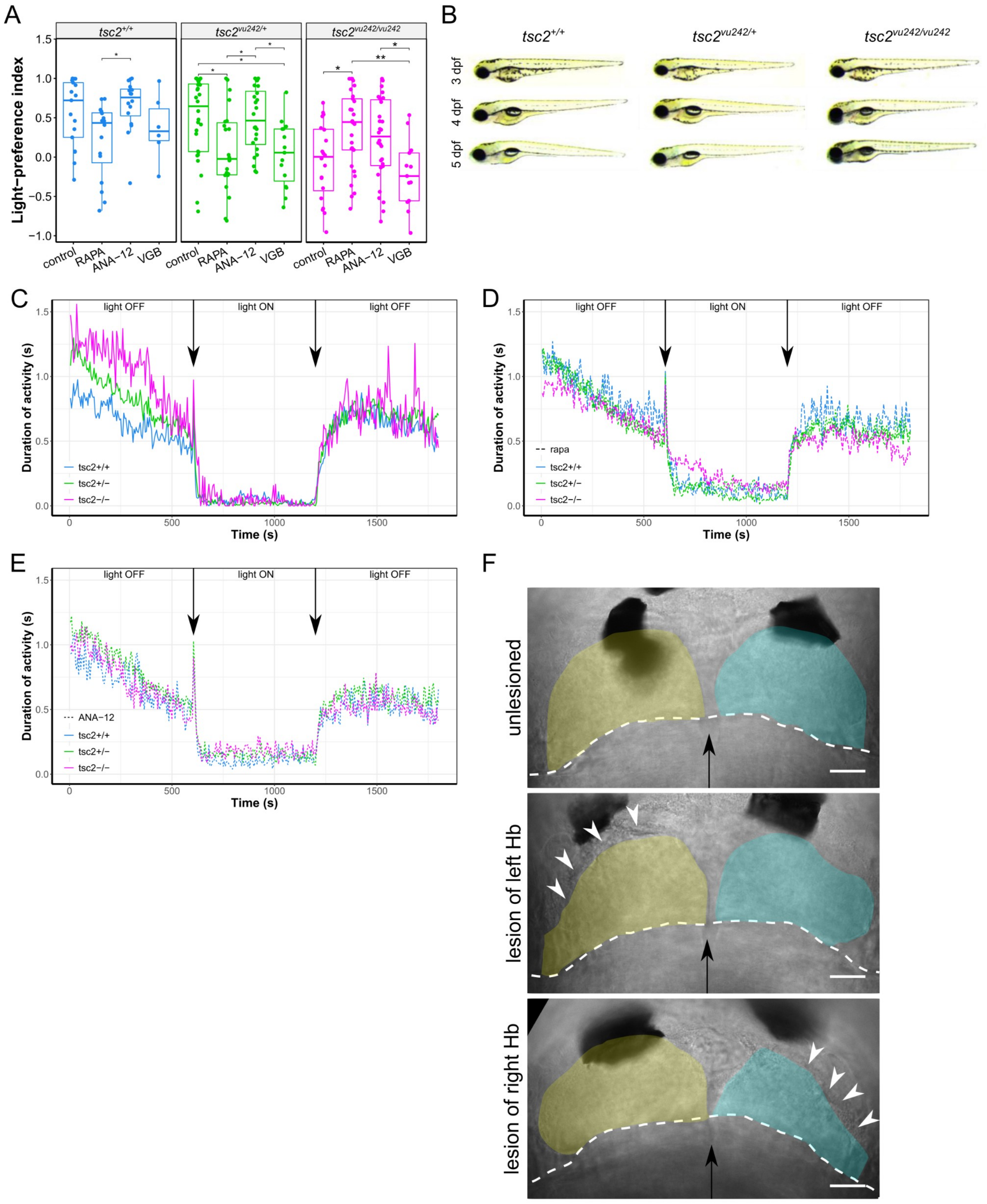

Supplementary Figure S1, related to Figure 1. Light-preference test in *tsc2*^{vu242} fish.

(A) Light-preference index of *tsc2*$^{+/+}$ sibling controls and *tsc2*$^{vu242/+}$ mutants with comparison statistics between treatments (*tsc2*$^{+/+}$: p = 0.013 for ANA-12 *vs.* RAPA, the rest – *ns*; *tsc2*$^{vu242/+}$: p = 0.036 for control *vs.* RAPA, p = 0.027 for control *vs.* VGB, p = 0.036 for ANA-12 *vs.* RAPA, p = 0.036 for ANA-12 *vs.* VGB).

(B) Gross morphology of *tsc2*^{vu242} fish. dpf – days post-fertilization

(C) Mean activity over time for *tsc2*^{vu242} fish after sudden changes in light conditions, showing proper responses to light changes: hyperactivity in the dark phases and freezing behavior in the light phase.
(D) Mean activity over time for *tsc2*^{vu242} fish treated with rapamycin after sudden changes in light conditions, showing proper responses to light changes: hyperactivity in the dark phases and freezing behavior in the light phase.
(E) Mean activity over time for *tsc2*^{vu242} fish treated with ANA-12 after sudden changes in light conditions, showing proper responses to light changes: hyperactivity in the dark phases and freezing behavior in the light phase.
(F) Exemplary bright-field images of habenulas morphology after lesions (taken at 4 dpf). The white dashed lines represent boundary between the optic tectum and the frontbrain, white arrows point the sites of the lesion, the black arrows point the midbrain boundary (between hemispheres). Left habenulas were colored in yellow and the right habenulas – in blue. Black patches are melanophores. Scale bars 20 μm.

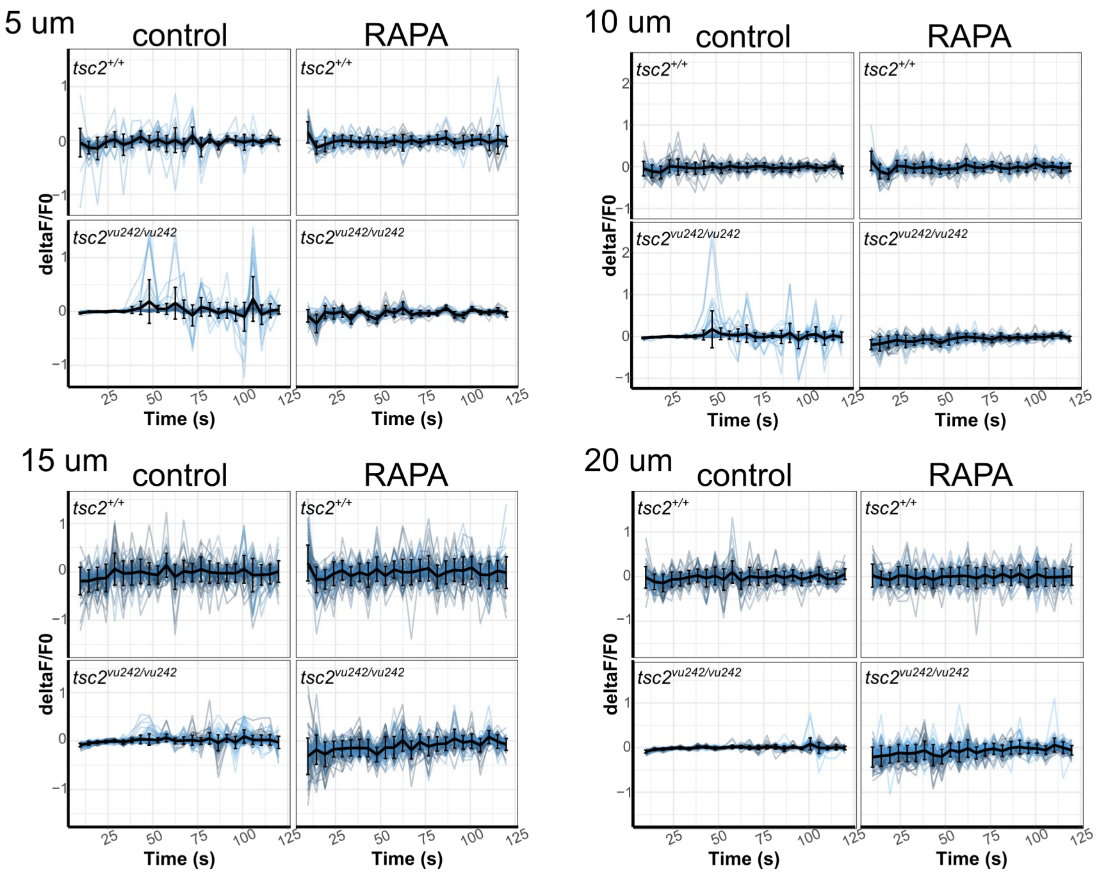

Supplementary Figure S2, related to Figure 3. Neuronal activity in LdHb of $tsc2^{vu242/vu242}$ fish and their wt siblings.

Neuronal activity change over time in the $tsc2^{+/+}$ and $tsc2^{vu242/vu242}$ LdHb at 5, 10, 15, and 20 µm from the top. In black – mean with SD.

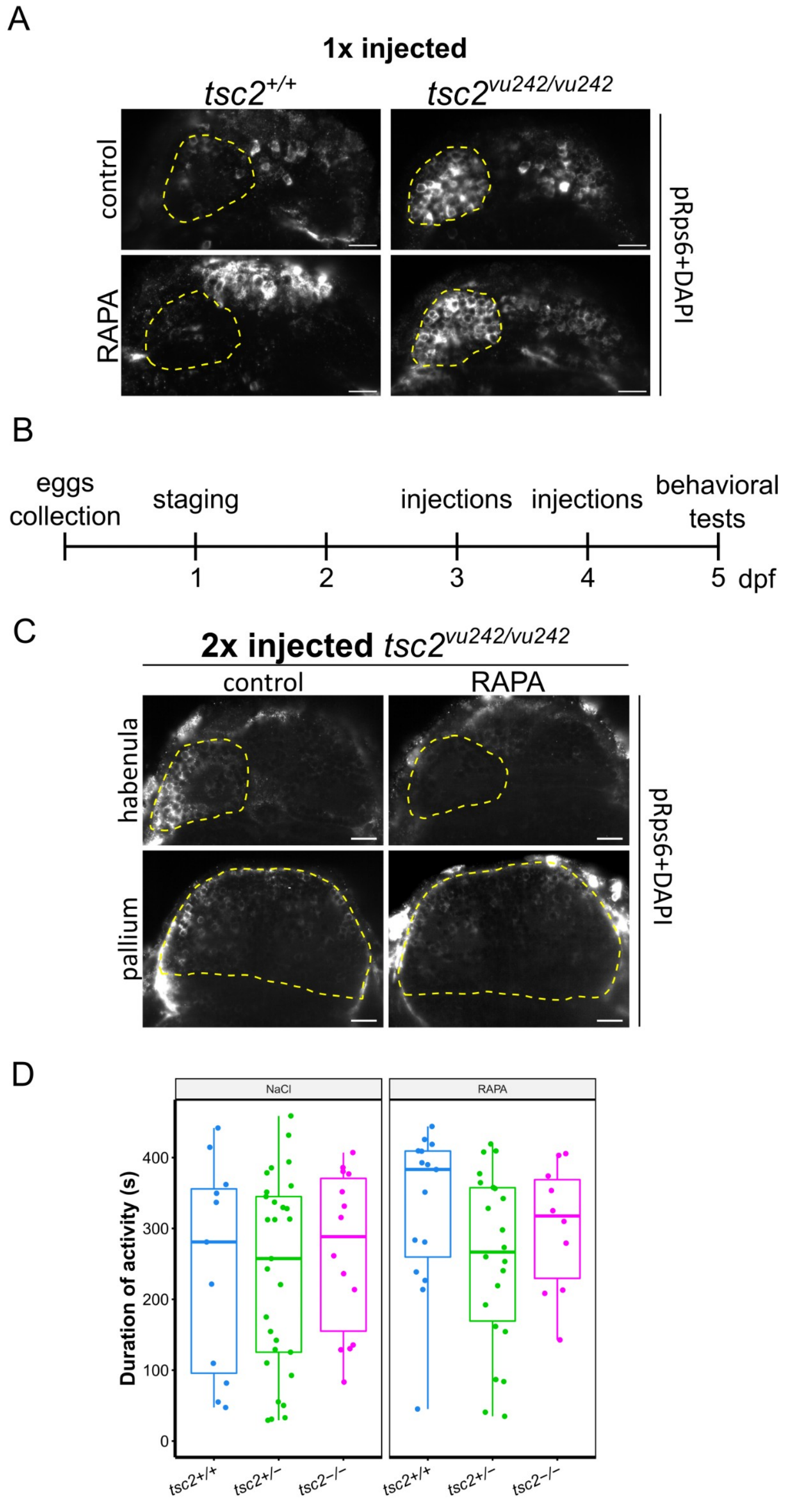

Supplementary Figure S3, related to Figure 4. Rapamacin injections to the left habenula and afferent connectivity of LdHb in *tsc2*^{vu242} fish.

(A) Exemplary images of P-Rps6-immunofluorescence intensity at the habenulae of the *tsc2*$^{vu242/vu242}$ fish and their wild-type *tsc2*$^{+/+}$ siblings after injections with rapamycin or NaCl at 4 dpf only. Injection at 4 dpf did not change P-Rps6 levels in the left habenula of the *tsc2*$^{vu242/vu242}$ mutant fish. Scale bars, 20 μm.

(B) Timeline of the experiment of rapamycin injections into the habenula.

(C) Exemplary images of P-Rps6-immunofluorescence intensity of the *tsc2*$^{vu242/vu242}$ fish after injections with rapamycin or NaCl at 3 dpf and 4 dpf depicting sections through habenulae and pallium. Injections at two consecutive days decreased the P-Rps6 levels in the left habenula of the *tsc2*$^{vu242/vu242}$ mutant fish, but did not change the P-Rps6 levels in the pallium. Scale bars, 20 μm.

(D) Cumulative activity during the light-preference test calculated for *tsc2*^{vu242} fish injected with rapamycin or NaCl at 3 dpf and 4 dpf.
